# An orthodontic perspective on Larsen syndrome

**DOI:** 10.1186/s12903-021-01454-x

**Published:** 2021-03-10

**Authors:** Madoka Yasunaga, Hiroyuki Ishikawa, Kenichi Yanagita, Sachio Tamaoki

**Affiliations:** 1 Section of Orthodontics, Department of Oral Growth and Development, Fukuoka Dental College, 2-15-1 Tamura, Sawara-ku, Fukuoka, 8140193 Japan; 2 Executive Trustee, Educational Institution, Fukuoka Gakuen, 2-15-1 Tamura, Sawara-ku, Fukuoka, 8140193 Japan; 3 Pediatric Dentistry, Fukuoka Children’s Hospital, 5-1-1 Kashiiteriha, Higashi-ku, Fukuoka, 8130017 Japan

**Keywords:** Larsen syndrome, Skeletal morphology, Craniofacial anomalies, Cephalometric analysis

## Abstract

**Background:**

Larsen syndrome (LS) is a rare disorder of osteochondrodysplasia. In addition to large-joint dislocations, craniofacial anomalies are typical characteristics. In this report, we performed orthodontic analyses, including skeletal and occlusal evaluations, to examine whether the craniofacial skeletal morphology leads to the craniofacial anomalies in LS.

**Case presentation:**

A 5 year old Japanese girl who was clinically diagnosed with LS was referred to the orthodontic clinic in the Fukuoka Dental College Medical and Dental Hospital because of a malocclusion. Clinical findings at birth were knee-joint dislocations, equinovarus foot deformities, and cleft soft palate. The patient showed craniofacial anomalies with hypertelorism, prominent forehead, depressed nasal bridge, and flattened midface. To evaluate the craniofacial skeletal morphology, cephalometric analysis was performed. In the frontal cephalometric analysis, the larger widths between bilateral points of the orbitale were related to hypertelorism. The lateral cephalometric analysis revealed the midface hypoplasia and the retrognathic mandible. These findings were responsible for the flattened appearance of the patient’s face, even if the anteroposterior position of the nasion was normal. Her forehead looked prominent in relation to the face probably because of the retrognathic maxilla and mandible. Both the study model and the frontal cephalometric analysis indicated constriction of the upper and lower dental arches. The posterior crossbite facilitated by the premature contacts had developed in association with the constriction of the upper dental arch.

**Conclusions:**

This patient had some craniofacial anomalies with characteristic appearances in LS. It was evident that the underlying skeletal morphology led to the craniofacial dysmorphism.

## Background

Larsen syndrome (LS) was first described by Loren J. Larsen in 1950. It is a very rare genetic or nongenetic (sporadic) osteochondrodysplasia [1]. LS affects approximately one in 100,000 newborn children each year [2]. LS is clinically represented by various features showing multiple congenital dislocations of the hip, knee, and elbow joints, equinovarus, or equinovalgus foot deformities, cylindrically shaped fingers, spinal anomalies—including scoliosis and cervical kyphosis—hearing loss caused by malformations of the ear ossicles, and characteristic craniofacial abnormalities [1, 3–5]. The diagnosis of LS is based on these clinical findings [6]. LS is genetically heterogeneous, and consists of autosomal recessive or autosomal dominant disorders caused by respective mutations in the *CHST3*, *B4GALT7*, and *GZF1* genes [7], or in the *FLNB* gene [8–11]. Genetic testing of the presence of mutations in these genes provides a useful adjunct to the diagnosis of LS patients with atypical or milder clinical manifestations [9, 10, 12]. The prognosis in the autosomal dominant form is relatively favorable than that in the recessive form if patients are treated with orthopedic surgery, physical therapy, fixation in plaster, or procedures used to treat the various symptoms associated with LS. However, in the autosomal recessive form, the clinical phenotype is associated with a higher mortality than the autosomal dominant form [9]. Intelligence in individuals with LS is usually unaffected [10].

Cleft palate was also reported in 23–50% of LS patients [6, 13, 14]. Characteristic facial features considered as craniofacial anomalies include a prominent forehead (frontal bossing), flattening of the bridge of the nose (depressed nasal bridge), wide-set eyes (ocular hypertelorism), and flattened midface (midface hypoplasia) [1, 3–5, 15–20]. However, the relationship between the craniofacial skeleton and facial anomalies is unclear because there are only a few reports related to the evaluation of the craniofacial skeleton in LS [15–17]. In addition to these anomalies, some reports have also referred to the presence of dental anomalies in LS, such as hypodontia, supernumerary teeth, microdontia, and malocclusions [15–20], but dental disturbances remain controversial.

In this report, we presented a Japanese girl diagnosed with LS and examined whether craniofacial skeletal and dental arch morphology lead to craniofacial anomalies and dental disturbances based on the orthodontic examinations.

## Case presentation

A 5 year old Japanese girl who had previously been diagnosed with LS was referred to the orthodontic clinic in the Fukuoka Dental College Medical and Dental Hospital in Fukuoka, Japan, because she had posterior crossbite. She is the second child with healthy parents who have no family history of genetic disorders. The diagnosis of LS was based on clinical findings. At birth, she had knee joint dislocations, equinovarus foot deformities, and a cleft of the soft palate only with no extension to the hard palate, alveolus, or lip (type 1 of Veau’s classification [21]). Operations for dislocations and equinovarus deformities were respectively performed at the ages of 4 and 9 months. Furlow’s palatoplasty was performed for the soft palate repair when she was 1.7 years old. She had not undergone any orthodontic treatment including presurgical orthodontics. The Enjoji scale [22], recorded at the age of four, revealed delays of social behavior and emotional and language development.

**Fig. 1:**
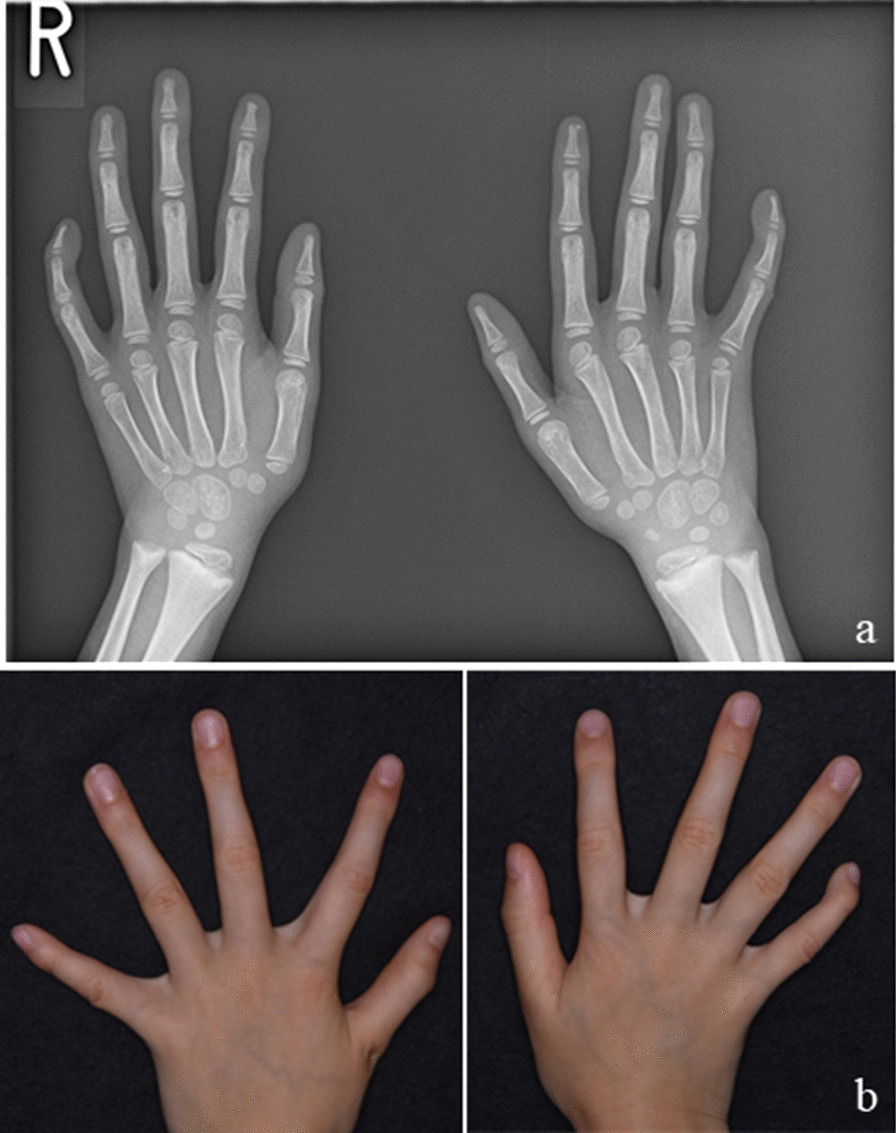
A hand-wrist radiograph and a photo of hands of the patient. **a** A hand–wrist radiograph was used to evaluate the skeletal age. **b** Cylindrically shaped fingers

**Fig. 2:**
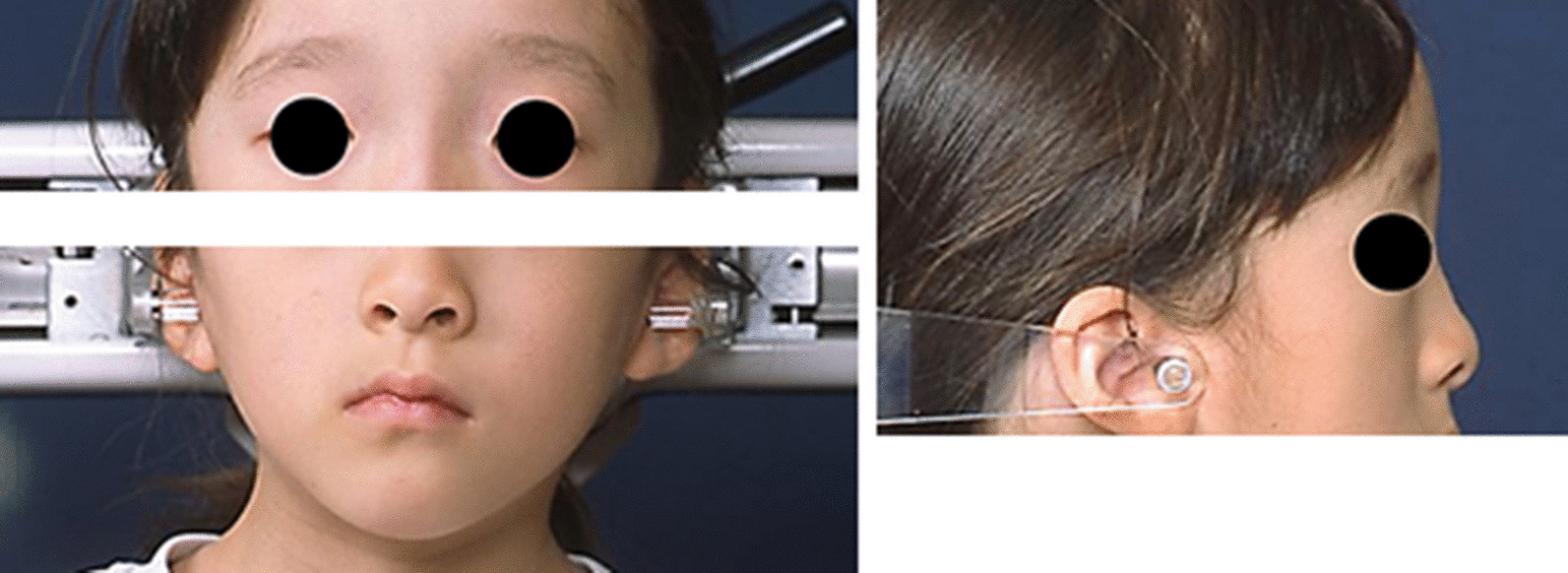
Facial photos of the studied patient showing hypertelorism, a prominent forehead, depressed nasal bridge, and a flat face

She underwent orthodontic examinations, including general, facial and intraoral examinations, lateral and frontal cephalometric analyses, and a study model examination for orthodontic diagnosis. On general examination, her height and weight were 112.3 cm and 15.1 kg, respectively. These were below the reference for the 75th and 10th percentile in her age and sex group, respectively. The hand–wrist radiograph was used to examine her skeletal maturity (Fig. 1a). The patient's skeletal age was matched at 5.3 years by the TW2 RUS method applied to the Japanese population, whereas the Greulich–Pyle Atlas method estimated the corresponding age at 5.5 years [23]. Based on these methods, her skeletal age almost matched her chronological age of 5.6 years. She had long cylindrically shaped fingers (Fig. 1b). Dysmorphic facial features were noted, including hypertelorism, a prominent forehead, depressed nasal bridge, and a flattened midface. Her facial profile was straight. Her chin point deviated toward the right side from the facial midline (Fig. 2).

**Fig. 3:**
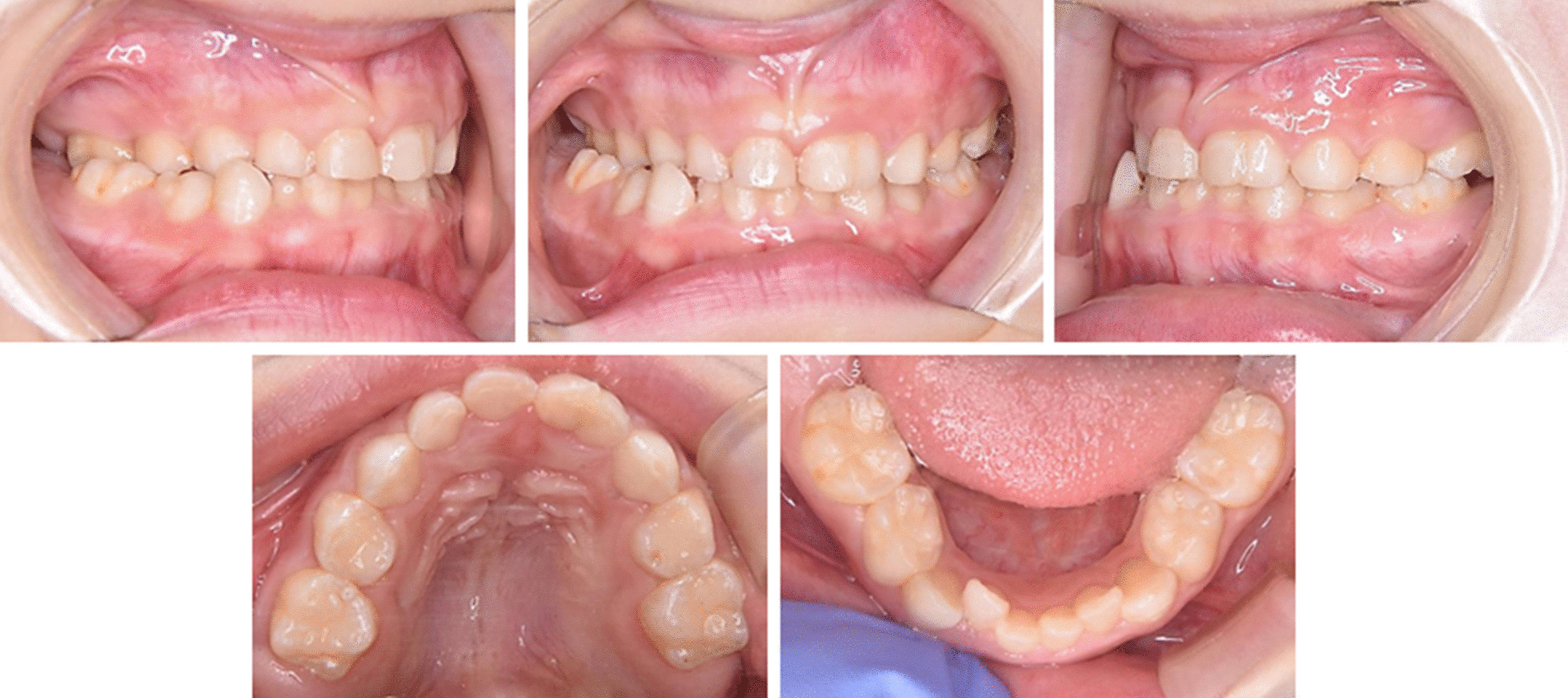
Intraoral photos of the studied patient. Absence of spaces in the upper dentition, dental crowding in the lower dentition, and posterior crossbite in the right primary molars are shown

Intraoral examination revealed the complete primary dentition with an overjet of 2.0 mm, an overbite of 2.0 mm, and mesial step-type terminal planes on both sides. There were no spaces around the fused tooth with the left deciduous central and lateral incisors in the upper dentition, while the lower dentition exhibited anterior crowding and mesial rotation of the first deciduous molars on both sides. On the right side, posterior crossbite was observed resulting from a functional lateral shift of the mandible owing to the premature contacts between the maxillary and mandibular right primary molars. In centric relation prior to the mandibular shifting, the mandibular dental midline was nearly consistent with the facial midline. In centric occlusion, the maxillary and mandibular dental midlines deviated 1.5 mm to the left and 2.0 mm to the right relative to the facial midline, respectively. As a result, the upper and lower dental midline discrepancy was 3.5 mm (Fig. 3).

**Fig. 4:**
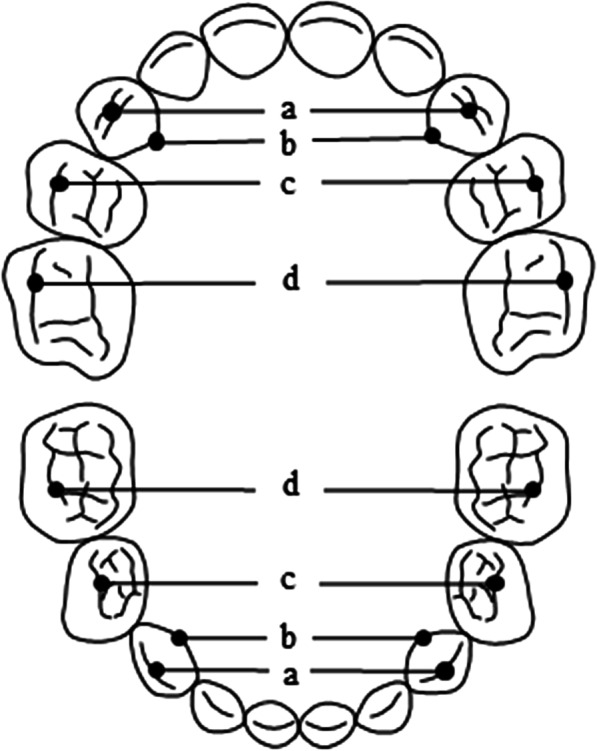
Dental arch width measurements. **a** Inter-cuspal width of primary canines (Cc-Cc). **b** Inter-palatal/lingual cervical line of canines (CL-CL). **c** Inter-buccal groove of primary first molars (D-D). **d** Inter-buccal groove of primary second molars (E-E)

On study models examination, the arch widths between the primary canines, and the first and second molars were measured to compare them with the standard values obtained from Japanese children with normal primary occlusions (Fig. 4 and Table 1) [24]. In the case of the primary canine, all the distances of the bilateral tips and the bilateral palatal or lingual cervical lines were measured. In the cases of the primary molars, the distances between the bilateral buccal grooves of the first and second molars were also measured. On the maxillary and mandibular dentitions, all the arch width measurements were significantly smaller than one standard deviation (SD) below the Japanese norms matched for the patient’s age.

**Table 1:** Study model examination

Measurements	Present case	Japanese children* [24]	
		Mean	SD
Maxilla			
Cc-Cc	23.6	30.73	1.71
C_L_-C_L_	17.5	25.48	1.65
D-D	32.6	39.85	1.68
E-E	37.5	46.10	1.77
Mandible			
Cc-Cc	19.0	23.84	1.34
C_L_-C_L_	14.5	19.55	1.40
D-D	24.0	33.48	1.73
E-E	32.0	38.62	1.51

Dental arch width measurements are in millimeters

*The means and SDs were given as mixed sex values for Cc-Cc, CL-CL, and D-D, whereas those were given as girl’s values for E-E.

**Fig. 5:**
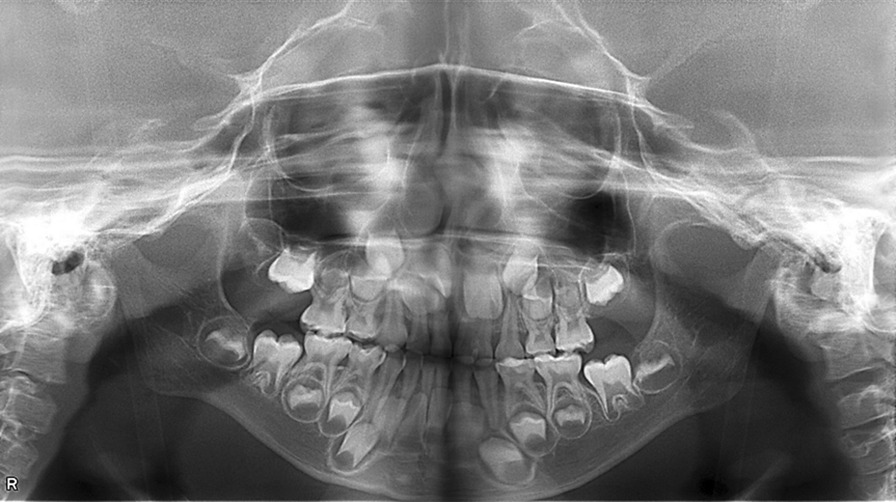
Panoramic radiograph showing the absence of the maxillary left second molar and lateral incisor

The panoramic radiograph showed that the maxillary left lateral incisor was congenitally missing. The tooth germ of the maxillary left second molar was not present. The tooth crown of the unerupted, maxillary right central incisor was rotated and was close to that of the maxillary right lateral incisor. The fused tooth with the left deciduous central and lateral incisors showed the union of tooth crowns by the dentin and two roots with independent root canals (Fig. 5).

**Fig. 6:**
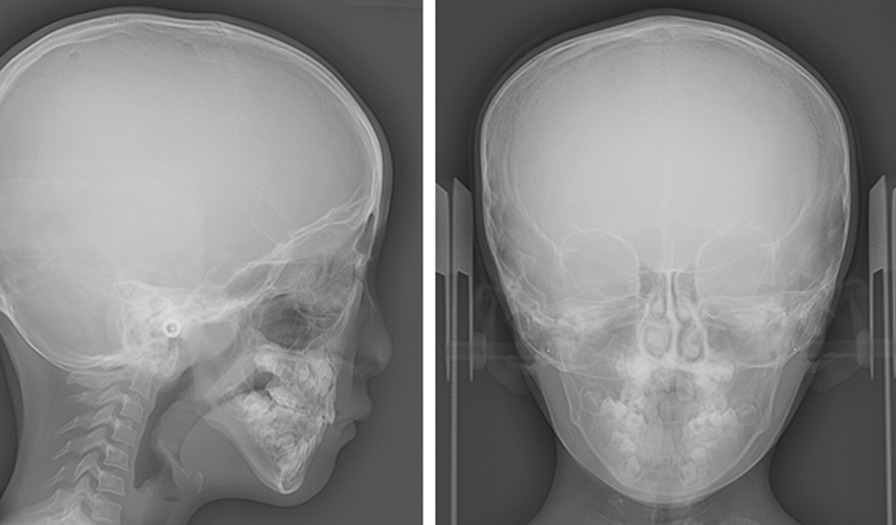
The lateral and frontal cephalograms

**Fig. 7:**
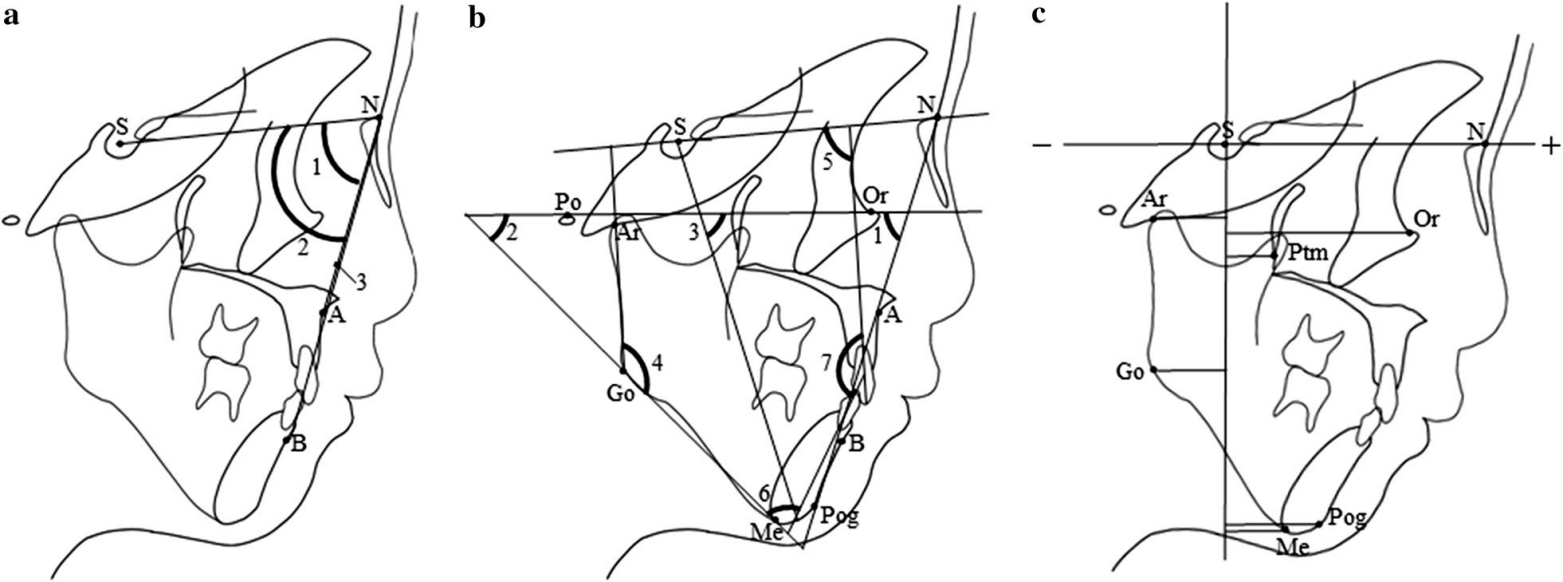
Lateral cephalometric landmarks and measurements. **a** (1) SNA, (2) SNB, and (3) ANB. **b** (1) Facial angle, (2) Frankfort-mandibular plane angle (FMA), (3) Y-axis, (4) Gonial angle, (5) U1 to SN, (6) L1 to mandibular plane angle (L1 to Mp), (7) Interincisal angle (S, sella; N, nasion; A, point A; B, point B; Po, porion; Or, orbitale; Go, gonion; Pog, pogonion; Me, menton and Ar, articulare). c. Facial depth representation (S, sella; N, nasion; Or, orbitale; Ptm, pterygomaxillary fissure; Pog, pogonion; Me, menton; Go, gonion and Ar, articulare)

Figure 6 shows the lateral and frontal cephalogram of the patient. Cephalograms were taken in a standard manner with a magnification ratio of 1.1. Figure 7a, b shows the conventional cephalometric analyses. The results are given in Table 2. These indicated the posteriorly positioned maxilla and mandible (SNA, SNB, and facial angle), skeletal Class III jaw relationship (ANB), steep mandibular plane (FMA) with the large Gonial angle, and growth tendency of the mandible (Y-axis) toward the postero-inferior direction. Inclination of the maxillary primary incisors was within the normal range (U1 to SN), while the mandibular primary incisor inclined more lingually than the norm (L1 to Mandible) [25].

**Table 2:** Lateral cephalometric analysis

	Present case	Japanese girl [25, 26]	
		Mean	SD
Angular measurement (in degrees)			
SNA	68.5	80.09	3.43
SNB	68.0	76.04	3.47
ANB	+ 0.5	+ 4.67	1.73
Facial angle	71.0	84.50	3.24
FMA	44.5	29.50	3.40
Y-axis	74.0	61.51	3.39
Gonial angle	139.0	129.95	5.26
U1 to SN	89.0	87.19	6.51
L1 to Mp	68.0	85.71	4.11
Interincisal angle	150.0	148.40	9.50
Facial depth measurement (in millimeters)			
N	63.0	62.0	2.43
Or	43.5	51.1	2.66
Ptm	11.5	14.8	2.63
Pog	23.5	38.3	5.67
Me	14.5	30.2	5.53
Go	− 17.5	− 14.0	3.62
Ar	− 18.0	− 15.7	2.21

Figure 7c shows the facial depth measurements used in this study. The horizontal positions of the six landmarks in relation to a line perpendicular to the S–N line that passed through sella, were measured based on the method proposed by Ono [26]. The results of the facial depth measurements were shown in Table 2. The depths measured on Or, PTM, Pog, Me, and Ar were smaller than one SD below the Japanese norms matched for the patient’s age and sex, while the depth of the nasion was within the normal range [26]. For the posterior part of the mandible, the facial depths measured on Go were close to one SD below the norms. These indicated that the orbitale, the maxilla, and mandible were positioned posteriorly relative to the anterior cranial base.

**Fig. 8:**
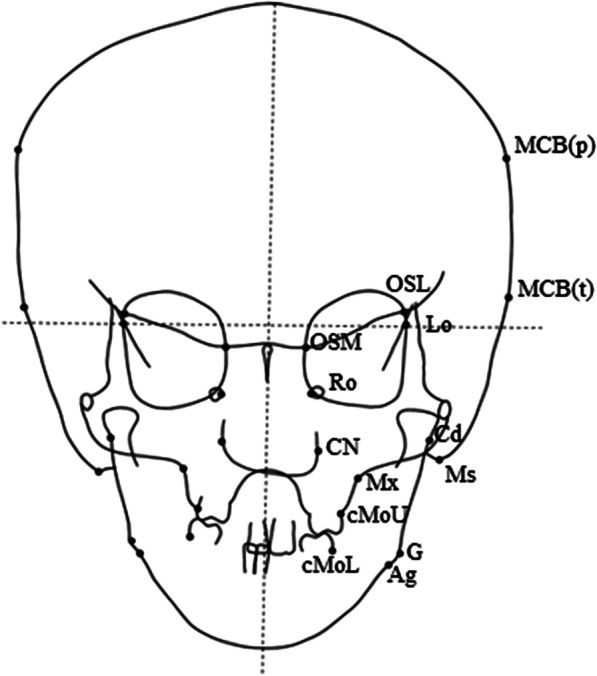
Frontal cephalometric landmarks and measurements (MCB(p), maximum cranial breadth (parietal bone); MCB(t), maximum cranial breadth (temporal bone); Lo, latero-orbitale; OSM, cross-point between anterior cranial fossa and orbital outlines; OSL, cross-point between the superior margin of the lesser wing of the sphenoid bone and orbital outline; Ro, foramen rotundum; CN, outmost point on the nasal cavity outline; Ms, mastoidale; Mx, maxillare; cMoU, cervical line of primary molars on the maxilla; cMoL, cervical line of primary molars on the mandible; Cd, condylion; Go, gonion and Ag, antegonial notch)

The frontal cephalometric analysis was performed as shown in Fig. 8. The facial midline was drawn through the Crista Galli and perpendicular to the line that passed through the latero-orbitale (Lo) bilaterally. For each of the 14 landmarks used, the distance to the facial midline was measured on both sides. The facial widths were obtained based on the addition of the bilateral distances measured on each landmark [26]. Table 3 shows the results of the facial width measurements. Compared with the Japanese norms matched for the patient’s age and sex, the cranial width measurements taken at MCB(p), Lo, OSM, OSL, and CN yielded values that exceeded the Japanese norms by one SD. The widths measured on the Mx, cMoU, and cMoL, were smaller than those of the Japanese norms, and indicated narrow maxillary basal arch and maxillary and mandibular dental arch widths.

**Table 3:** Frontal cephalometric facial width measurements

Measurement	Present case	Japanese girl [26]	
		Mean	SD
MCB(p)	155.0	144.5	7.84
MCB(t)	152.5	150.7	6.65
Lo	88.5	84.8	3.33
OSM	25.0	21.5	1.89
OSL	89.5	79.1	3.61
Ro	27.5	30.7	3.42
CN	30.0	26.7	1.72
Ms	107.0	103.0	5.66
Mx	55.0	62.0	2.60
cMoU	45.0	50.9	2.20
cMoL	45.5	48.2	2.20
Cd	101.0	99.0	5.06
Go	84.5	83.5	4.19
Ag	79.0	76.6	3.90

Facial width measurements are in millimeters

Based on the examinations performed, an orthodontic diagnosis of unilateral posterior crossbite due to a functional lateral shift of the mandible, a skeletal Class III jaw relationship, narrow maxillary and mandibular dental arches, mandibular anterior mild crowding, and congenital missing of the maxillary left lateral incisor and the maxillary left second molar was made. The proposed treatment plan included (1) the maxillary dental arch expansion to eliminate the premature contacts between the maxillary and mandibular right primary molars and to improve the lateral shift of the mandible, (2) observation of the jaw growth and occlusal development with potential options of the maxillary protraction and the mandibular dental arch expansion, and (3) a fixed appliance treatment for the permanent dentition. We initiated the treatment with the use of the removable expansion plate for the maxillary dental arch expansion. No obvious problems have been observed in treatment response of the patient so far.

## Discussion and conclusions

In this case, we performed cephalometric analyses to examine how the craniofacial anomalies were linked to craniofacial skeletal morphologies. The frontal cephalometric analysis exhibited skeletal characteristics that related to hypertelorism, and yielded increased widths measured at OSM, OSL, and Lo, compared with the Japanese norms. Larger widths measured between bilateral points of the orbitale were directly linked to the broadly spaced eyes.

The lateral cephalometric analysis revealed the midface hypoplasia and the retrognathic mandible. Several studies have attempted to assess the significance of the flat face or the posterior position of the maxilla in LS with lateral cephalometric analyses [15–17]. Some reports noted that the SNA angles of LS patients were smaller than those of the norms. These findings are indicative that the maxilla was posteriorly positioned. For the mandible, there are studies that yielded smaller SNB angles compared with the norms [15–17]. The cephalometric analysis in this study showed that both the SNA and SNB angles were significantly smaller than those of Japanese girls. The facial depth measurements revealed the posterior positions of Or, Pog, and Me. These findings indicated that the orbital area, the maxilla, and the mandible were located posteriorly relative to the anterior cranial base. The skeletal features appear to result in a flat face that is one of typical facial anomalies in LS. Even though the anteroposterior position of the nasion was normal, her forehead looked prominent relative to the face, probably owing to the posterior position of the midface and the mandible. The cephalometric analyses provided new details on the relations of the craniofacial appearances and the skeletal morphology in LS.

Table 4 summarizes the oral findings in case reports of LS [15–20]. In this case, dental abnormalities contain hypodontia and contracted dental arches. The maxillary left second molar and the lateral incisor seemed to be congenitally missing in this case. Some reports revealed that the prevalence of the maxillary lateral incisor hypodontia was higher in patients with cleft palate compared with the non-cleft population [27]. In the present case, the congenitally missing teeth of the maxillary lateral incisor may be related to the cleft palate. However, the association of the second molar hypodontia with the cleft palate is unclear. The frequency of hypodontia (in the absence of any disorders) was reported to range from 1.6% and 10.09% [28, 29], while four out of 19 LS individuals including the present case and 18 cases in previous reports [1, 10, 15–19] had missing teeth attributed to congenital etiologies. Whether this is a typical dental symptom in LS is still debated. Despite the fact that the mechanisms responsible for the missing teeth remain unclear, some of the factors that facilitate osteochondrodysplasia may be related to the impairment of the tooth germs and may disrupt tooth formation [30]. The patient exhibited an overjet of 2.0 mm and an overbite of 2.0 mm, while previous studies reported a variety of overjet and overbite findings in LS. Constricted upper and lower dental arches were observed as the outcomes of the study model and frontal cephalometric analysis, leading to the absence of spaces in the upper dentition and to a dental crowding in the lower dentition. Furthermore, posterior crossbite in the right primary molars facilitated by premature contacts were associated with constricted upper dental arches. Previous reports revealed two cases of unilateral and two cases of bilateral posterior crossbites as well as one case with undetermined bite sites. However, no prior reports described the interactions between the posterior crossbites and contracted dental arches. This patient underwent an operation of the soft palate cleft when she was 1.7 years old. The incident rate of cleft palate in LS ranges between 23 and 50% [6, 13, 14]. In cleft palate patients, the contracted maxillary dental arch was related to the underdevelopment of the maxilla owing to the postsurgical scar tissue formation on the palates [31]. Given that the operative method used in this patient was the Furlow palatoplasty without periosteal detachment, the formed scar was considered to be small [31, 32]. In addition, the cleft was limited to the soft palate. A weak relationship may exist between the contracted maxillary dental arch and the cleft palate in this case. Considering that several case reports showed posterior crossbites [15–18, 20] or a high-arched palate [33] in LS, maxillary dental arch constrictions can often occur in LS, irrespective of whether it induces premature contacts or not. As shown in this case, the lateral shift of the mandible resulted from the premature contacts that caused deviation of the chin point, and may have facilitated the formation of facial asymmetries during growth.

**Table 4:** Summary of information of oral findings in seven cases of Larsen syndrome presented in this study and in previous reports [15–20]

	Tsang et al. [15]	Chien et al. [18]	Kawahara et al. [16]	Percin et al. [19]	Kozaki et al. [20]	Sajnani et al. [17]	Present case
Age	15	29	8	14	8	8	5
Sex	Female	Female	Male	Female	Female	Male	Female
Number of abnormal teeth	11 CMT	None	4 CMT	2 CST	ND	2 CMT	2 CMT
Cleft lip and/or palate	Cleft left lip and palate	ND	Cleft palate	Cleft palate	Cleft soft palate	None	Cleft soft palate
Incisor relationship	O.J.: − 6 mm anterior crossbite	ND	O.J.: − 2 mm O.B.: + 5 mm anterior crossbite	ND	Edge-to-edge bite	Anterior open bite	O.J.: + 2 mm O.B.: + 2 mm
Posterior cross bite	Unilateral: left	Found*	Bilateral	ND	Unilateral: right	Bilateral	Unilateral: right

*CMT* congenital missing teeth, *CST* congenital supernumerary teeth, *O.J.* overjet, *O.B.* overbite, *ND* no description

*Posterior cross bite with no description as to whether unilateral or bilateral

This case report revealed that the underlying craniofacial skeletal morphology leads to the craniofacial dysmorphism in LS.

## Data Availability

All data generated or analyzed during this study are included in this published article.

## Abbreviation

LS: Larsen syndrome
